# Detailed One-Year Follow-Up in Left Bundle Branch Area Pacing: Echocardiography, Natriuretic Peptide, Electrical Parameters and Complications

**DOI:** 10.3390/jcm13061532

**Published:** 2024-03-07

**Authors:** Maciej Fularz, Przemysław Mitkowski

**Affiliations:** 1 Department of Cardiology, University of Zielona Góra, ul. Zyty 28, 65-046 Zielona Góra, Poland; 2 1st Department of Cardiology, Poznań University of Medical Sciences, ul. Długa 1/2, 61-848 Poznań, Poland; przemyslaw.mitkowski@usk.poznan.pl

**Keywords:** complications, echocardiography, electrical parameters, left bundle branch area pacing, natriuretic peptide

## Abstract

**Background**: LBBAP is a promising method of cardiac pacing. Data on some follow-up details are still limited. We aimed to evaluate LBBAP over a one-year follow-up period. **Methods**: The studied population consisted of 110 patients who underwent LBBAP device implantation (93 for bradycardia indications, 17 for CRT). Echocardiography and NT-proBNP measurement were performed before the procedure and after one year. Electrical parameters, complications and some other conditions that required attention were noted during the observation period. **Results**: In total, 89 patients completed the one-year follow-up. NT-proBNP and echocardiographic parameters (LVEF, left ventricular end-diastolic dimension, left atrium dimension) improved after the one-year follow-up, especially in the patients with CRT indications, but also in the bradycardia patients. The independent predictors of a positive heart function response were higher baseline NT-proBNP and the presence of either RBBB or LBBB. The electrical parameters were satisfactory but a slight raise in the pacing threshold was observed at subsequent control visits. Major complications occurred in 4.5% of patients in the acute phase and in 4.4% during the follow-up (the most common was dislodgement of the non-LBBAP lead). One patient is presumed to have developed pacing-induced cardiomyopathy. The most frequent mild complications were intraprocedural RBBB (9.1%) and conversion to deep septal pacing during the follow-up period (5.5%). In addition, 4.4% of the patients required individual programming of the pacing output to maintain the optimal type of capture. **Conclusions**: The impact of LBBAP device implantation on cardiac function parameters is positive in a wide range of indications, especially in patients with RBBB or LBBB. The prevalence of complications is relatively high but major complications are rarely associated with LBBAP issues.

## 1. Introduction

Left bundle branch area pacing (LBBAP) has developed rapidly over the last few years. The feasibility and safety of this promising pacing modality have been shown not only in small to medium studies [1,2,3,4] but also in large multicenter registers [5]. The main advantage of LBBAP is the provision of better ventricular synchrony than in conventional right ventricular pacing (RVP) [6]. The superior outcomes of LBBAP compared to RVP in heart failure hospitalization and all-cause mortality rates, as well as in various parameters of cardiac function, have been shown in a few studies [7,8,9]. The mechanism underlying these results is fast activation of the left ventricle by left bundle branch (LBB) fibres or via quick capture of the left side of the septal myocardium. This may not only prevent the development of pacing-induced cardiomyopathy [10,11], but may also improve ventricular synchrony, restoring impaired conduction in patients with left bundle branch block (LBBB). Individuals with right bundle branch block (RBBB) may benefit from a reduction in right ventricular activation delay due to the capture of the septal myocardium. These resynchronizing effects ensure the efficacy of LBBAP in cardiac resynchronization therapy (CRT). Improvement in the NYHA functional class, left ventricular ejection fraction (LVEF), left ventricular end-diastolic diameter (LVEDD) and left ventricular volumes have been shown in recent studies [12,13,14,15,16]. However, detailed data on the impact of LBBAP on the parameters of cardiac function in bradycardia patients, especially with preserved LVEF, as well as on some follow-up issues, are still limited.

The aim of this study was to evaluate LBBAP during a one-year observation period. We assessed echocardiographic response, changes in the N-terminal prohormone of the brain natriuretic peptide (NT-proBNP) serum level and electrical parameters. Moreover, we indicated the factors associated with better outcomes in heart function parameters. Furthermore, we conducted a detailed analysis of acute and follow-up complications and conditions that required special attention or management.

## 2. Materials and Methods

### 2.1. Study Design and Population

The studied population consisted of 110 patients who underwent successful LBBAP device implantation between March 2021 and June 2022. No preselection was applied—LBBAP was performed as the primary approach for all pacing indications. There were no exclusion criteria besides the inability to achieve LBBAP. All consecutive patients who had received an LBBAP device and had been operated on by the authors within the indicated period of time were included in the study. Patients were observed for a one-year period. Outpatient control visits were performed 1, 6 and 12 months after implantation.

### 2.2. LBBAP Lead Implantation

A lumenless (SelectSecure 3830, Medtronic, Minneapolis, MN, USA) lead was delivered by a corresponding fixed shape catheter (C315-HIS, Medtronic) to the right ventricle and allocated towards the interventricular septum. The initial position was determined using fluoroscopy and pace mapping. Regarding fluoroscopy, we used a simplified method similar to Zhang et al., targeting a wide area of the septum from approximately 2 cm behind the tricuspid summit to about half of the distance to the apex [17]. To confirm the appropriate position, unipolar tip pacing was performed—we followed the criterion of QRS polarity discordance in leads aVL (positive) and aVR (negative). Recording of the His bundle potential was not required. After determining the initial site, lead rotations were started. The depth of the penetration was monitored by observation of fixation beats [18] and we repeated the unipolar tip pacing until the terminal R-wave in lead V1 appeared. Electrocardiographic and electrical measurements were then performed. If the parameters were acceptable, the delivery catheter was removed.

### 2.3. Device Programming

The pacemakers were programmed conventionally with a few exceptions. Firstly, decisions on whether to promote intrinsic conduction or ventricular pacing in patients with preserved atrioventricular conduction were individualized. In patients with a narrow native QRS complex and normal PR interval, longer AV delays were set to avoid pacing. In individuals with bundle branch block or a prolonged PR interval, we programmed short AV delays to achieve the resynchronization effect. Secondly, in the case of dissimilarity in the capture thresholds of the septal myocardium and LBB, the ventricular output was set manually to ensure conduction system capture or minimum adapted amplitude was increased accordingly. The CRT devices were programmed individually to achieve maximal narrowing of the QRS complexes. This included adjustment of the AV delay and choosing a bipolar (to achieve anodal capture) or unipolar configuration of the LBBAP lead. In the case of a prominent RBBB pattern during LBBAP, additional pacing from the right ventricular lead was used.

### 2.4. Data Collection

Echocardiography and NT-proBNP serum level measurement were performed prior to implantation and 12 months after the procedure (at the third control visit). Vivid T8 and T9 (GE Healthcare, Chicago, IL, USA) ultrasound machines were used. To assess the echocardiographic response, LVEF, LVEDD and left atrial dimension (LAD) were measured in a two-dimensional presentation. We also assessed the distance from the base of the anterior mitral cusp to the tip of the LBBAP lead in an apical four-chamber view. Heart function response was defined as meeting at least two of the following criteria: (1) a reduction of at least 50% in the NT-proBNP level, (2) an increase of at least 5 percentage points in LVEF, (3) a decrease of at least 5 mm in LVEDD and (4) a decrease of at least 5 mm in LAD.

Standard electrical parameters (capture threshold, sensing and impedance) were measured intraprocedurally, the next day and at every control visit (1, 6 and 12 months after implantation). Complications were noted at any time they were revealed. Pacing-induced cardiomyopathy (PICM) was defined as a drop in LVEF of at least 10 percentage points in the case of a high ventricular pacing burden after the exclusion of alternative causes (coronary artery disease, valvular heart disease, alcohol addiction, etc.). The electrocardiographic parameters required in LBBAP were measured at a high sweep speed with digital callipers (R-wave peak time in lead V6 (V6-RWPT), V6-V1 interpeak interval, paced and native QRS duration). The criteria for LBB capture were used according to recent EHRA consensus [19].

### 2.5. Statistical Analysis

In the case of normally distributed variables, the results were reported as means and standard deviations; otherwise they were reported as medians and interquartile ranges. Student’s *t*-test for paired samples or the Wilcoxon signed-rank test if necessary were used to compare the NT-proBNP and echocardiographic parameters before implantation and after 12 months. To identify the factors associated with changes in the cardiac function parameters, Student’s *t*-test for independent samples, the Mann–Whitney U test and correlation coefficients (Pearson’s and Spearman’s) were applied. Univariable and multivariable logistic regression analyses were performed to find the predictors of heart function response. To compare the electrical parameters at subsequent visits, a Friedman test was conducted. Statistical analysis was performed using PSPP 1.6.2 software. A two-sided *p*-value < 0.05 was considered statistically significant.

## 3. Results

### 3.1. Baseline Characteristics

The baseline characteristics of the studied group including demographic data, comorbidities, pacing indications and the type of device are shown in Table 1. In total, 8 out of 17 implantations for cardiac resynchronization therapy (CRT) indications were primary, while 9 were upgrades (5 in patients with implantable cardioverter-defibrillator and intraventricular conduction delay, 3 in patients with PICM and 1 in a patient with dysfunctional biventricular pacing).

### 3.2. Completion of Follow-Up Period

Overall, 89 of the 110 patients fully completed the planned follow-up period. A total of 21 patients did not finish the 12-month observation period for the following reasons: death (9 cases), not showing up for planned visit (10 cases), Twiddler syndrome with dual dislodgement that required reimplantation of both leads (1 case) and infectious endocarditis in a patient with a haematological malignancy that required extraction of the device (1 case).

### 3.3. NT-proBNP Serum Level

The median NT-proBNP serum concentration decreased from 657 ng/pL to 286 ng/pL (Table 2). This effect was observed for both bradycardia and CRT indications; however, the improvement was greater in CRT patients (Table 3). Another factor linked with a more marked decrease in the NT-proBNP serum level was lower baseline LVEF (Spearman’s correlation coefficient 0.25, *p*-value = 0.04) and a higher baseline NT-proBNP serum concentration (Spearman’s correlation coefficient −0.57, *p*-value < 0.001). The percentage of ventricular pacing was not correlated with the change in the NT-proBNP level in bradycardia patients (Spearman’s correlation coefficient 0.04).

### 3.4. Echocardiographic Parameters

At 12 months after the LBBAP device implantation, we observed an increase in LVEF as well as a decrease in LVEDD and LAD in the entire group (Table 2). The improvement in LVEF and LAD was greater in CRT patients than in bradycardia patients (Table 3). In fact, the increase in LVEF was observed only in CRT patients. Nevertheless, an improvement in LVEF was noted in a subgroup of bradycardia patients with baseline LVEF < 50% (medians: baseline 45% and after one year 55%, *p*-value = 0.02), while LVEDD and LAD decreased in the entire bradycardia population. Changes in the tested parameters were similar in both types of bradycardia indication—atrioventricular block and sick sinus syndrome.

### 3.5. Factors Correlated with Greater Improvement

The parameters associated with the degree of improvement in NT-proBNP, LVEF, LVEDD and LAD are listed in Table 4. Among the baseline factors, the general trend was that a more impaired heart function before implantation was correlated with a greater improvement after the procedure. Patients with LBBB benefited more in terms of LVEF and LAD, while subjects with RBBB saw a greater decrease in LVEDD (also seen in patients with preserved LVEF). More detailed data on the changes in cardiac function parameters according to the native QRS complex type in the bradycardia group are provided in Table 5 (supplemented with information on ventricular pacing burden). Importantly, shorter V6-RWPT and confirmed left bundle branch capture were associated with a greater improvement in LVEDD in patients with bradycardia indications and a ventricular pacing burden > 40%. Greater narrowing of the QRS complex (or lesser widening) was related to a greater decrease in LAD. A more basal location of a tip (in terms of a shorter distance from the base of the anterior mitral cusp to the tip of the LBBAP lead) was connected with a larger increase in LVEF in CRT patients (Pearson’s correlation coefficient 0.69, *p*-value = 0.02). Some potentially significant effects might not have been identified because the power of testing in the CRT group was low due to the small number of patients (for example Pearson’s correlation coefficient between R-wave peak time in lead V6 and delta LVEF was −0.52 but the *p*-value was non-significant at 0.09).

### 3.6. Predictors of Response in Heart Function

Overall, 48.8% of the patients fulfilled the criteria for a positive heart function response, comprising 83.3% of those with CRT indications and 43.2% in the bradycardia group. In univariable analysis, the predictors of the response in heart function were CRT indications, lower baseline LVEF, the presence of RBBB or LBBB, a higher baseline NT-proBNP level, wider native QRS and lower delta in the QRS duration (Table 6). Multivariable analysis showed that two predictors were independent: the presence of RBBB or LBBB (but not nonspecific intraventricular conduction delay) and a higher baseline NT-proBNP level. In the bradycardia group, there were three independent predictors: the presence of RBBB or LBBB (odds ratio 5.95, *p*-value = 0.01), baseline NT-proBNP level (odds ratio 1.32 per 500 pg/mL, *p*-value = 0.05) and baseline LAD (odds ratio 2.07 per 5 mm, *p*-value = 0.03).

### 3.7. Electrical Parameters

The pacing parameters were satisfactory (Table 7). The capture threshold was highest during implantation (median 1.0 V), lowest the next day (median 0.4 V) and then gradually increased (median 0.8 V after one year). The maximal observed ventricular capture threshold in an individual patient was 2.25 V × 0.4 ms, but it remained stable and did not require the repositioning of a lead. Interestingly, the correlation between the capture threshold at implantation and after 12 months was negligible (Spearman’s correlation coefficient 0.20, *p* = 0.06). R-wave sensing was lowest at the time of the procedure (median 11 mV) and then was constant (median 16 mV). Impedance was highest during implantation, then decreased slightly and stabilized after one month.

### 3.8. Complications

**Figure 1:**
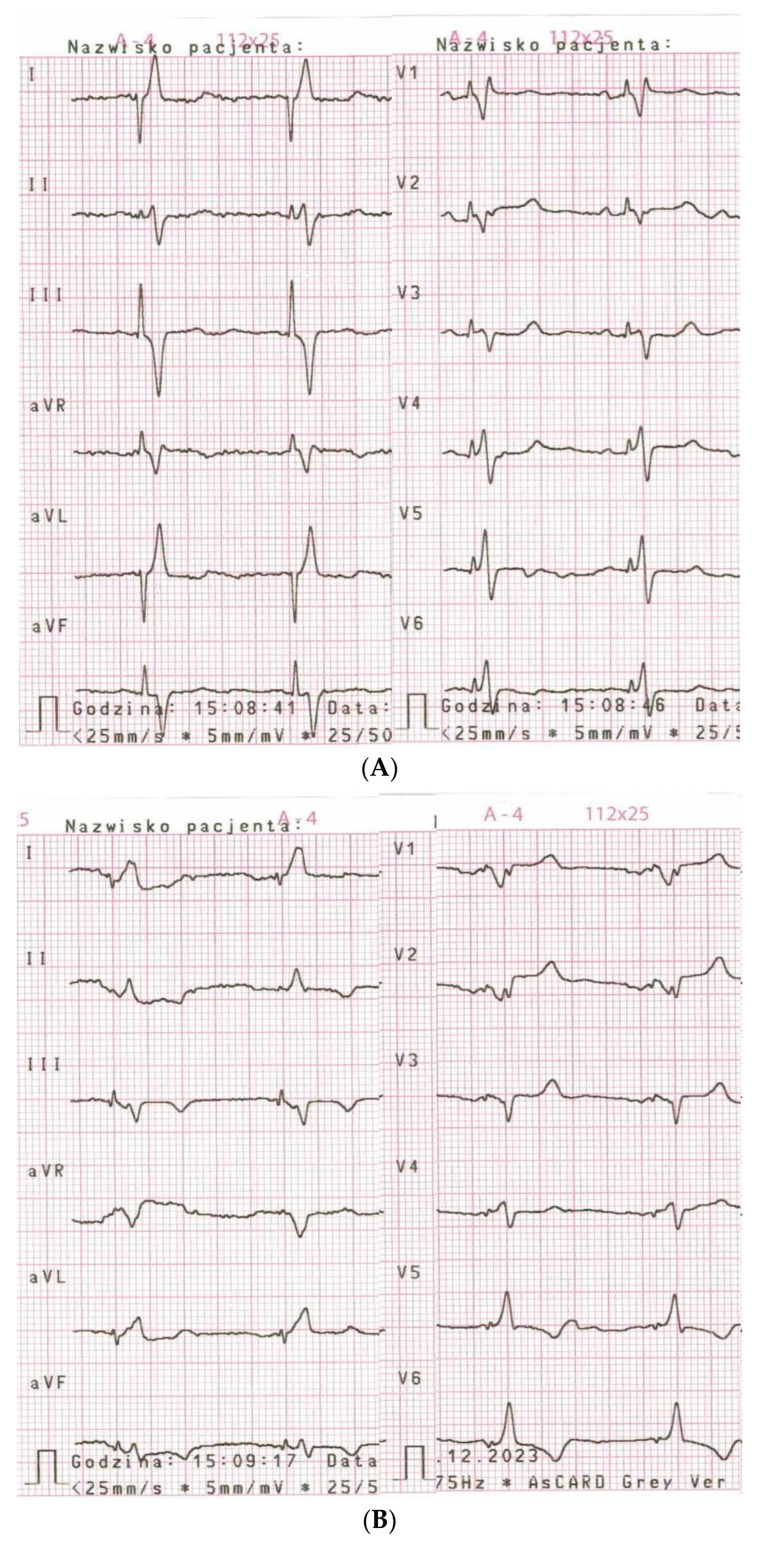
Example of strong dissimilarity in capture threshold of left bundle branch and myocardium identified in follow-up period. (**A**). Non-selective left bundle branch capture was observed at pacing output of at least 2.5 V × 0.4 ms; (**B**). Myocardial capture was observed at pacing output from 0.75 V × 0.4 ms to 2.25 V × 0.4 ms.

The observed complications are listed in Table 8. Most of the major complications were general and not directly connected with LBBAP. However, one case of probable PICM was noted. The patient, with permanent atrial fibrillation and bradycardia, but without clinical symptoms of heart failure before implantation, presented with peripheral oedema and exertional dyspnoea six months after the procedure. The ventricular pacing burden was 100%. One year after implantation, LVEF decreased from a baseline of 55% to 39% and NT-proBNP increased from 2162 pg/mL to 4330 pg/mL. The symptoms were partially alleviated after the administration of oral diuretics. Coronary artery disease was excluded. The patient had a notably long V6-RWPT (101 ms), but the criteria for LBBAP were met during the entire follow-up period. On the other hand, all three patients who underwent LBBAP due to PICM during RVP received a significant benefit (LVEF increased from 30%, 33% and 35% to 40%, 42% and 45%, respectively). Among the mild complications, the most frequent was iatrogenic RBBB, which occurred during 10 procedures and persisted after one year in three cases. Acute perforation to the left ventricle cavity occurred in five patients, without further consequences. Conversion to deep septal pacing (loss of LBBAP) occurred in five cases and was probably caused by micro-dislocations. It is noteworthy that in four patients, despite a low general capture threshold, programming of a higher output was required to maintain non-selective LBB capture (Figure 1).

## 4. Discussion

The major findings of this study are as follows: (1) LBBAP device implantation leads to a notable improvement in heart function parameters after a one-year follow-up, both in CRT and bradycardia patients, (2) a higher baseline NT-proBNP level and the presence of either RBBB or LBBB predict particularly positive heart function responses, (3) the electrical parameters of LBBAP leads are satisfactory and stable, (4) most severe complications of LBBAP device implantation are not related to the LBBAP lead, (5) the most frequent complications are iatrogenic RBBB, which usually subsides, and conversion to deep septal pacing (loss of LBBAP) and (6) some patients require switching off or modification of the auto-threshold algorithms to maintain an optimal type of capture.

The efficiency of LBBAP in CRT patients has already been proven in small to medium studies. An echocardiographic response has been shown in the general CRT population, in patients with RBBB and in those with PICM indications, as well as in those with LBBAP-optimized CRT [12,13,14,15,16]. Our results are consistent with these findings, showing a significant improvement in NT-proBNP level, LVEF and LAD in the non-selected CRT population, despite the small group studied. We revealed a strong correlation between the distance from the base of the anterior mitral cusp to the tip of the LBBAP lead and a change in LVEF. This finding suggests that proximal left bundle branch capture in the basal part of the septum may enhance the effect of CRT.

Importantly, a lower but still significant improvement in the NT-proBNP level and some echocardiographic end-points was also noted in the bradycardia patients. Although LVEF did not increase in this group, the positive response in the NT-proBNP level, LVEDD and LAD may suggest that the left ventricular end-diastolic pressure decreased. The mechanism underlying this improvement in the bradycardia patients seems to be complex. Subjects with a high-grade atrioventricular block benefit from an increase in heart rate and restoration of atrioventricular synchrony without significant impairment in intraventricular synchrony [6]. However, an improvement was also noted in patients with sick sinus syndrome. Several factors may contribute to this effect. The first is release from bradycardia (by atrial pacing or occasional ventricular pacing). Moreover, patients with bundle branch block or first-degree atrioventricular block may benefit from the resynchronizing effect of LBBAP (pacing was promoted in 29% of patients with sick sinus syndrome). Finally, better general treatment due to regular follow-up visits may also influence the results. However, quantification of the contributions of individual mechanisms is hard to assess without a direct comparison of LBBAP and RVP.

Bednarek et al. [10] showed that LVEF increased in bradycardia patients with baseline LVEF below 50%, while it was stable in individuals with preserved LVEF, and they concluded that LBBAP prevents PICM in patients with preserved LVEF and improves left ventricle function in subjects with depressed LVEF. Our results are, in general, consistent with these findings. However, on the one hand, the outcomes of the present study suggest that patients with preserved LVEF also receive a benefit in heart function (improvement in NT-proBNP, LVEDD and LAD), but on the other hand it should be emphasized that we noted a case of presumed PICM. Since we found that shorter V6-RWPT, confirmed left bundle branch capture and greater narrowing of the QRS complex were related to better outcomes in some echocardiographic end-points, particular attention may be paid to achieving satisfactory results in these parameters, especially in patients with any evidence of a heart function impairment, because this population is particularly prone to PICM [20] (the patient who developed PICM had a relatively high baseline NT-proBNP level).

Our results showed that the presence of either RBBB or LBBB predicts a positive heart function response in the unselected LBBAP population as well as in the bradycardia indication group (with an even greater odds ratio). The possible explanation of this phenomenon is the dualistic nature of LBBAP. LBB capture provides quick activation of the left ventricle, while concomitant myocardial septal capture ensures faster propagation to the right ventricle than in native conduction with RBBB. This effect may be further enhanced by ring capture [14]. An improvement in ventricular synchrony in the case of nonspecific intraventricular conduction delay, usually associated with ventricular hypertrophy or scar, remains a challenge.

It has already been shown that the electrical parameters in LBBAP are satisfactory and stable in short-term follow-up [5,11]. We noticed that the pacing parameters significantly improved between implantation and the next day. Regarding the capture threshold, this constantly increased after the initial decrease. This suggests that the capture threshold still requires long-term observation. Special attention should be paid to identifying a possible dissimilarity in the capture thresholds of the myocardium and left bundle branch. Four patients from the studied group required individual programming of the pacing output to maintain non-selective LBB capture, since the LBB pacing threshold was notably greater than the myocardial one and the default auto-threshold settings led to a loss of it and, in consequence, to an increase in V6-RWPT and QRS duration, as well as to worse ventricular synchrony [6]. Therefore, electrocardiographic evaluation of QRS morphology at different pacing outputs at control visits is needed to reveal potential transitions of capture types and provide the adequate programming of devices.

The cumulative number of complications observed in our study seems to be relatively high. However, most of the major complications were general and not directly related to the LBBAP lead. The case of possible PICM has been noted, but it would presumably also have occurred if RVP had been used instead of LBBAP. This thesis may be supported by our observation that three patients with baseline PICM improved after upgrading from RVP to LBBAP. Moreover, the prevalence of PICM during RVP is more than 10% [20]. On the other hand, most of the minor complications are linked with LBBAP. We reported iatrogenic RBBB in 9.1% of patients intraprocedurally and in 3.3% after one year. The prevalence of this complication may be even higher—19.9% and 6.3%, respectively [11]—while septal perforation occurs in up to 14.4% of patients, usually without further repercussions [19]. The most frequent complication in the follow-up period was the conversion to deep septal pacing, which was noted in 5.5% of cases. Nevertheless, the electrocardiographic effect of this modality is still better than in RVP.

## 5. Limitations

The main limitations of this study are as follows: (1) the low number and heterogeneity of the CRT population, (2) 19% of the patients did not complete the follow-up period (8% of the patients had died), which may have led to an overestimation of LBBAP’s benefits, (3) a one-year follow-up is still a relatively short period of time, (4) the lack of comparison with RVP excludes a clear assessment of the contribution of the individual mechanisms underlying the heart function improvement and (5) the results of the heart function response predictors analysis depend on the definition of heart function response.

## 6. Conclusions

The implantation of an LBBAP device seems to be appropriate in a wide range of CRT and bradycardia indications, particularly in patients with RBBB or LBBB. Although an improvement in NT-proBNP and echocardiographic parameters was noted in the entire group, the LBBAP outcomes varied significantly between individuals. Since not only the baseline factors but also some intraprocedural outcomes (shorter V6-RWPT, narrowing of QRS complex, confirmed LBB capture, more basal location of a tip) are possibly related to a better response in some parameters of cardiac function, it may be particularly important to achieve satisfactory results during implantation. The complications connected with the LBBAP lead are usually mild but relatively frequent. Electrocardiographic monitoring of the LBB capture threshold is needed to provide adequate programming of the LBBAP device.

## Figures and Tables

**Table 1:** Baseline characteristics of the studied group.

**General**	
Age, years, mean (SD)	74.9 (8.7)
Female sex, *n* (%)	43 (39.1)
Primary implantation, *n* (%)	101 (91.8)
**Pacing indications**	
Atrioventricular block, *n* (%)	56 (50.9)
Sick sinus syndrome, *n* (%)	37 (33.6)
Cardiac resynchronization therapy, *n* (%)	17 (15.5)
**Device type**	
LBBAP, *n* (%)	98 (89.1)
LBBAP + ICD, *n* (%)	10 (9.1)
LBBAP + RVP, *n* (%)	2 (1.8)
**Comorbidities**	
Heart failure, *n* (%)	33 (30)
Coronary artery disease, *n* (%)	29 (26.4)
Hypertension, *n* (%)	89 (80.1)
Atrial fibrillation, *n* (%)	47 (42.7)
Diabetes, *n* (%)	37 (28.2)
Chronic kidney disease, *n* (%)	15 (13.6)
COPD, *n* (%)	10 (9.1)

Abbreviations: COPD, chronic obstructive pulmonary disease; ICD, implantable cardioverter-defibrillator; LBBAP, left bundle branch area pacing; RVP, right ventricular pacing; SD, standard deviation.

**Table 2:** Comparison of heart function parameters before implantation of LBBAP device and after 12-month follow-up.

Parameter		NT-proBNP, pg/mL, Median (IQR)	LVEF, %, Median (IQR)	LVEDD, mm, Median (IQR)	LAD, mm, Mean (SD)
Entire group	Pre-implant	657 (250–1432)	55 (49–60)	51 (47–54)	42.1 (5.3)
	After 12 months	286 (162–806)	57 (52–62)	47 (44–51)	38.7 (5.7)
	*p*-value	<0.001	0.02	<0.001	<0.001
Bradycardia	Pre-implant	450 (228–1193)	58 (55–60)	50 (46–53)	41.4 (4.8)
	After 12 months	267 (149–719)	59 (54–64)	46 (43–51)	38.4 (5.0)
	*p*-value	0.008	0.24	<0.001	<0.001
CRT	Pre-implant	1397 (1258–3934)	30 (25–34)	57 (55–70)	46.0 (6.1)
	After 12 months	731 (286–1744)	40 (32–43)	57 (48–65)	40.4 (8.4)
	*p*-value	0.02	0.008	0.18	0.002

Abbreviations: CRT, cardiac resynchronization therapy; IQR, interquartile range; LAD, left atrial dimension; LBBAP, left bundle branch area pacing; LVEDD, left ventricular end-diastolic dimension; LVEF, left ventricular ejection fraction; NT-proBNP, N-terminal prohormone of brain natriuretic peptide; SD, standard deviation.

**Table 3:** Comparison of changes in heart function parameters between CRT and bradycardia group.

Parameter	Delta ^a^ NT-proBNP, pg/mL, Median (IQR)	Delta ^a^ LVEF, %, Mean (SD)	Delta ^a^ LVEDD, mm, Mean (SD)	Delta ^a^ LAD, mm, Mean (SD)
Bradycardia	−77 (−473 to 73)	1.1 (7.4)	−2.4 (4.7)	−2.9 (3.4)
CRT	−990 (−2607 to −485)	8.8 (9.1)	−2.6 (6.7)	−5.6 (4.8)
*p*-value	0.006	0.005	0.79	0.04

Abbreviations: CRT, cardiac resynchronization therapy; IQR, interquartile range; LAD, left atrial dimension; LVEDD, left ventricular end-diastolic dimension; LVEF, left ventricular ejection fraction; NT-proBNP, N-terminal prohormone of brain natriuretic peptide; SD, standard deviation; ^a^ Delta was calculated as value after 12 months minus baseline value.

**Table 4:** Factors associated with greater improvement in investigated parameters.

Factor	Parameters with Greater Improvement (Statistics)
CRT indication	NT-proBNP; LVEF; LAD (see in Table 3)
Lower baseline LVEF	NT-proBNP (r_s_ = 0.25, *p* = 0.04); LVEF (r_s_ = 0.58, *p* <0.001)
Presence of native LBBB	LVEF (increase: 6.1% vs. 1.2%, *p* = 0.03); LAD (decrease: 5.2 mm vs. 2.9 mm, *p* = 0.03)
Presence of native RBBB	LVEDD (decrease: 5.3 vs. 1.8 mm, *p* = 0.02 in entire group; 5.2 vs. 2.0 mm, *p* = 0.03 in patients with LVEF > 50%)
Higher baseline NT-proBNP	NT-proBNP (r_s_ = 0.57, *p* < 0.001)
Higher baseline LVEDD	LVEDD (r_s_ = 0.35, *p* = 0.002)
Higher baseline LAD	LAD (r = 0.24; *p* = 0.04)
Wider native QRS	LAD (r_s_ = 0.23, *p* = 0.047)
More basal location of a tip	LVEF (r = 0.69; *p* = 0.02)—in CRT indication
Confirmed LBB capture	LVEDD (decrease 3.5 mm vs. increase 0.9 mm; *p* = 0.02)—in bradycardia patients with percentage of VP > 40%
Greater QRS narrowing	LAD (r = 0.25; *p* = 0.03)
Shorter paced V6-RWPT	LVEDD (r = 0.34; *p* = 0.03)—in bradycardia patients with percentage of VP > 40%

Abbreviations: CRT, cardiac resynchronization therapy; LAD, left atrial dimension; LBBB, left bundle branch block; LVEDD, left ventricular end-diastolic dimension; LVEF, left ventricular ejection fraction; NT-proBNP, N-terminal prohormone of brain natriuretic peptide; r, Pearson’s correlation coefficient; r_s_, Spearman’s correlation coefficient; RBBB, right bundle branch block; V6-RWPT, R-wave peak time in lead V6.

**Table 5:** Comparison of heart function parameters before implantation of LBBAP device and after 12-month follow-up in subgroups of bradycardia population according to native QRS type.

Parameter		NT-proBNP, pg/mL, Median (IQR)	LVEF, %, Median (IQR)	LVEDD, mm, Median (IQR)	LAD, mm, Mean (SD)	Number of Patients with VP > 40%, (%)
Narrow native QRS (<120 ms)	Pre-implant	532 (167–1536)	59 (55–61)	49 (46–53)	42.4 (4.7)	19 out of 40 (47.5)
	After 12 months	342 (156–849)	59 (56–62)	47 (44–51)	40.1 (5.0)	
	*p*-value	0.12	0.57	0.08	<0.001	
Broad native QRS (≥120 ms)	Pre-implant	414 (247–991)	55 (54–60)	51 (47–53)	40.3 (4.8)	29 out of 36 (80.6)
	After 12 months	244 (137–541)	57 (52–65)	46 (43–50)	36.6 (4.4)	
	*p*-value	0.03	0.35	0.001	<0.001	
RBBB **^a^**	Pre-implant	364 (243–1193)	58 (55–60)	51 (48–54)	39.3 (4.5)	13 out of 18 (72.2)
	After 12 months	206 (126–304)	60 (56–65)	45 (44–48)	35.1 (4.2)	
	*p*-value	0.02	0.32	0.004	<0.001	

Abbreviations: IQR, interquartile range; LAD, left atrial dimension; LBBAP, left bundle branch area pacing; LVEDD, left ventricular end-diastolic dimension; LVEF, left ventricular ejection fraction; NT-proBNP, N-terminal prohormone of brain natriuretic peptide; RBBB, right bundle branch block SD, standard deviation; VP, ventricular pacing burden; ^a^ Separate analyses of left bundle branch block and nonspecific intraventricular conduction delay were not conducted due to low number of patients in these subgroups.

**Table 6:** Predictors of heart function response.

Factor	Univariable Analysis OR (95% CI); *p*-Value	Multivariable Analysis OR (95% CI); *p*-Value
CRT indication	**6.61 (1.35–32.37); 0.02**	-
Baseline NT-proBNP (per 500 pg/mL)	**1.31 (1.01–1.10); 0.01**	**1.37 (1.08–1.74); 0.009**
Baseline LVEF (per 5%)	**0.69 (0.54–0.89); 0.004**	-
Baseline LVEDD (per 5 mm)	1.29 (0.91–1.82); 0.15	-
Baseline LAD (per 5 mm)	1.54 (0.97–2.44); 0.07	-
Presence of native LBBB	2.51 (0.83–7.64); 0.10	-
Presence of native RBBB	2.2 (0.72–6.78); 0.17	-
Presence of native NICD	0.84 (0.17–4.00); 0.82	-
Presence of RBBB or LBBB	**3.19 (1.27–8.04); 0.01**	**4.12 (1.46–11.65); 0.008**
Native QRS duration (per 10 ms)	**1.18 (1.01–1.37); 0.03**	-
Delta QRS ^a^ (per 10 ms)	**0.83 (0.69–0.99); 0.04**	-

Abbreviations: CI, confidence interval; CRT, cardiac resynchronization therapy; LAD, left atrial dimension; LBBB, left bundle branch block; LVEDD, left ventricular end-diastolic dimension; LVEF, left ventricular ejection fraction; NICD, nonspecific intraventricular conduction delay; NT-proBNP, N-terminal prohormone of brain natriuretic peptide; OR, odds ratio; RBBB, right bundle branch block; ^a^ Delta QRS was calculated as paced QRS duration minus native QRS duration.

**Table 7:** Electrical parameters at subsequent controls (bipolar set).

Parameter	Pacing Threshold, V, Median (IQR)	Sensing, mV, Median (IQR)	Impedance, Ohms, Mean (SD)
Intraprocedural	1 (0.8–1.2)	11 (7–14)	881 (187)
1 day after procedure	0.4 (0.4–0.5)	16 (8–22)	664 (117)
1 month after procedure	0.6 (0.5–0.8)	16 (11–22)	603 (105)
6 months after procedure	0.6 (0.6–0.9)	16 (11–22)	592 (108)
1 year after procedure	0.8 (0.6–0.9)	16 (11–16)	589 (96)
*p*-value	<0.001 ^a^	<0.001 ^b^	<0.001 ^c^

Abbreviations: IQR, interquartile range; SD, standard deviation; ^a^ In post hoc analysis, differences were significant between all measurements; ^b^ In post hoc analysis, differences were significant between intraprocedural and any other measurement; ^c^ In post hoc analysis, differences were significant between intraprocedural and any other measurement as well as between 1 day after procedure and any other point.

**Table 8:** Complications of LBBAP device implantation.

Acute Phase ^a^		Follow-Up ^b^	
Major complications (life-threatening or requiring intervention)			
Dislodgement of non-LBBAP lead	2.7%	Twiddler syndrome	1.1%
Pneumothorax	0.9%	Infectious endocarditis	1.1%
Pericardial effusion	0.9%	Pericardial effusion	1.1%
		Pacing-induced cardiomyopathy	1.1%
Patients with any major complication	4.5%	Patients with any major complication	4.4%
Minor complications and other conditions that required attention			
Intraprocedural iatrogenic RBBB	9.1%	Persistent iatrogenic RBBB	3.3%
Intraprocedural perforation to the left ventricle cavity	4.5%	Conversion to DSP (loss of LBBAP)	5.5%
		Mild dysfunction of non-LBBAP lead	3.3%
		High output (>2 V) required to maintain non-selective LBB capture	4.4%
		LBBAP lead capture threshold > 2 V	1.1%
Patients with any of aforementioned	13.6%	Patients with any of aforementioned	15.4%

Abbreviations: DSP, deep septal pacing; LBB, left bundle branch; LBBAP, left bundle branch area pacing; RBBB, right bundle branch block; ^a^ Percentages were calculated in relation to all 110 patients; ^b^ Percentages were calculated in relation to 91 patients (89 who completed one-year follow-up and 2 who did not due to Twiddler syndrome and infectious endocarditis).

## Data Availability

The data underlying this article will be shared on reasonable request to the corresponding author.
